# Water Safety Plan, Monochloramine Disinfection and Extensive Environmental Sampling Effectively Control *Legionella* and Other Waterborne Pathogens in Nosocomial Settings: The Ten-Year Experience of an Italian Hospital

**DOI:** 10.3390/microorganisms11071794

**Published:** 2023-07-13

**Authors:** Claudio Farina, Eleonora Cacciabue, Franca Averara, Nadia Ferri, Francesca Vailati, Gabriele Del Castillo, Antonello Serafini, Beatrice Fermi, Nicola Doniselli, Fabio Pezzoli

**Affiliations:** 1 Microbiology and Virology Laboratory, ASST “Papa Giovanni XXIII”, 24127 Bergamo, Italy; ferrnad@gmail.com (N.F.); fvailati@asst-pg23.it (F.V.); 2 Health Care Coordination Offices, ASST “Papa Giovanni XXIII”, 24127 Bergamo, Italy; ecacciabue@asst-pg23.it (E.C.); gdelcastillo@asst-pg23.it (G.D.C.); fpezzoli@asst-pg23.it (F.P.); 3 Department of Health Care Professions, ASST “Papa Giovanni XXIII”, 24127 Bergamo, Italy; faverara@asst-pg23.it; 4 Technical Services, ASST “Papa Giovanni XXIII”, 24127 Bergamo, Italy; aserafini@asst-pg23.it; 5 Sanipur S.p.A., 25020 Flero, Italy; b.fermi@sanipur.it (B.F.); n.doniselli@sanipur.it (N.D.); 6 ESCMID Study Group for Legionella Infections (ESGLI), 4001 Basel, Switzerland

**Keywords:** *Legionella*, Water Safety Plan, monochloramine, waterborne pathogens, prevention

## Abstract

*Legionella* contamination control is crucial in healthcare settings where patients suffer an increased risk of disease and fatal outcome. To ensure an effective management of this health hazard, the accurate application of a hospital-specific Water Safety Plan (WSP), the choice of a suitable water disinfection system and an extensive monitoring program are required. Here, the ten-year experience of an Italian hospital is reported: since its commissioning, Legionellosis risk management has been entrusted to a multi-disciplinary Working Group, applying the principles of the World Health Organization’s WSP. The disinfection strategy to prevent *Legionella* and other waterborne pathogens relies on the treatment of domestic hot water with a system ensuring the in situ production and dosage of monochloramine. An average of 250 samples/year were collected and analyzed to allow an accurate assessment of the microbiological status of water network. With the aim of increasing the monitoring sensitivity, in addition to the standard culture method, an optimized MALDI-ToF MS-based strategy was applied, allowing the identification of *Legionella* species and other relevant opportunistic pathogens. Data collected so far confirmed the effectiveness of this multidisciplinary approach: the fraction of positive samples never overcame 1% on a yearly basis and Legionnaires’ Disease cases never occurred.

## 1. Introduction

Since its debut and subsequent identification during the outbreak of Philadelphia in 1976 [1,2], *Legionella pneumophila* has shown itself as a serious risk for human health [3]. According to current knowledge, the *Legionella* genus includes over 70 species of aerobic, Gram-negative ubiquitous bacteria spread worldwide in both natural water bodies and artificial water distribution systems [4,5]: their growth and proliferation are particularly boosted in the latter engineered habitats, providing favorable water temperatures (25–45 °C), nutrients abundance and surfaces facilitating biofilm formation.

The *pneumophila* species, as well as other species of the genus, are responsible for the pathological conditions collectively known as legionellosis [6,7,8], comprising both Pontiac fever, a mild flu-like illness, and Legionnaire’s Disease (LD), the pneumonic and more severe form, that frequently lead to fatal outcomes [9,10].

The risk of *Legionella* infection represents a critical issue in particular for hospitals and other healthcare-related buildings [11,12,13]. Indeed, chronic lung diseases and, more in general, any condition associated with immunodeficiency can significantly contribute to increase individual susceptibility and prognosis severity [9]: patients of healthcare settings are thus generally prone to enhanced vulnerability. In addition, the practice of airways-related clinical procedures can increase the risk of exposure to such a kind of waterborne pathogens transmitted by inhalation of contaminated water droplets [14]. 

According to European data, the case fatality associated with hospital-acquired and healthcare-related infections, which accounts for the 8% of the total cases, increased from the general rate of 10% to 30–50% [15,16]. Recently, the Italian health authority Istituto Superiore di Sanità (ISS) reported that for 2021, the case–fatality ratio for hospital-acquired cases in Italy was even 40.5% [17]. 

Given the relevance of this opportunistic pathogen and of its consequences in terms of disease burden, it is no wonder that the Drinking Water Directive 2020 (2020/2184) introduced *Legionella* as a new parameter to be monitored to ensure a proper water quality, especially in the domestic water distribution systems of priority premises, and recommended a risk-based approach for its proper management.

In the last few years, an increasing amount of attention has been also addressed to other waterborne pathogens [18,19,20], both bacteria and fungi, that, similarly to *Legionella*, may represent a risk for human health, particularly for healthcare patients affected by chronical diseases or by immunocompromised states [21,22]. 

When dealing with microbiological water safety, it is important to consider that the construction and commissioning stages of a new building are characterized by an increased risk of microbial colonization and biofilm formation in water piping [23,24,25]: it is thus crucial, especially when dealing with healthcare settings, to rely on an effective strategy for *Legionella* (and other waterborne pathogens) risk management even before the opening of a new building, to properly keep the water distribution system status under control since its installation [26,27,28].

For this reason, in 2012, when moving from the old 1930s hosting building to a new, 997-bed structure, the Management of Papa Giovanni XXIII Hospital (Bergamo, Italy) appointed a dedicated multidisciplinary Working Group for the definition and implementation of a tailor-made Water Safety Plan (WSP) based on the principles stated by World Health Organization and national guidelines [4,29,30] to ensure a reliable strategy for legionellosis risk management even before commissioning.

The choice of the right water disinfection approach represents a crucial factor for such a strategy: it should ensure satisfactory results in terms of microbiological (and chemical) water safety while being fully compatible with the structure and composition of water distribution systems [31,32]. In this case, monochloramine was chosen for domestic hot water treatment: this chlorine-based disinfectant was used since the beginning of the last century by municipalities for the secondary disinfection of drinking water, but it was at its first applications for *Legionella* prevention in domestic hot water systems at that time [33]. 

In addition to the establishment of preventive and control measures, the Working Group drew up a strict protocol of self-checking, which was based on an extensive *Legionella* monitoring campaign. As analytical approach, an internal multistep procedure was adopted, combining a culture-based method with the downstream application of MALDI-ToF MS (Matrix-Assisted Laser Desorption/Ionization Time-of-Flight Mass Spectrometry) for *Legionella* identification at the species level. 

This approach allowed the monitoring not only of *Legionella* species but also of other waterborne opportunistic pathogens of nosocomial relevance, ensuring a more exhaustive control of the microbiological quality of water.

In the present work, the experience of Papa Giovanni XXIII Hospital from commissioning until 2022 is reported, with the aim of proving the importance of prevention and surveillance and the effectiveness of such strategies. 

## 2. Materials and Methods

### 2.1. Setting

**Figure 1:**
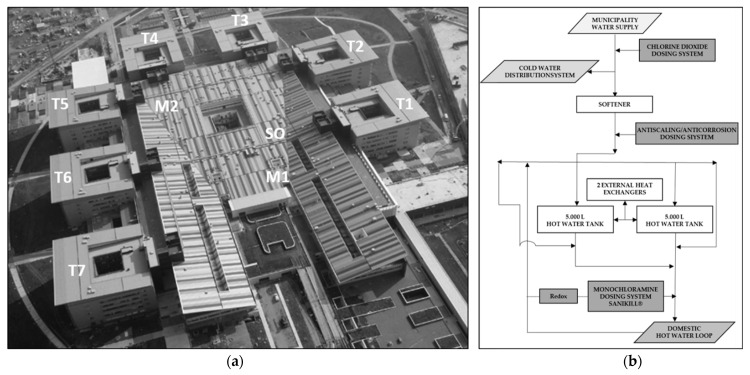
(**a**) Aerial view of the hospital depicting its structure and the positions of the domestic hot water production plants (T1–T7, M1, M2, SO); (**b**) schematic representation of the hot water production plants.

Papa Giovanni XXIII Hospital is one of the biggest healthcare facilities in Italy, with 997 beds distributed over 320,000 square meters. The hospital consists of 7 five-story towers and a three-story main building called “the Plate” (Figure 1). The towers host the wards while the main building hosts the emergency department, surgery rooms, ICUs, outpatient clinics, staff offices and laboratories. The pharmacy and some restaurants, bars and shops are also located at the lobby level. 

### 2.2. Piping and Water Treatment

Drinking water supplied by the local municipality (see Supplementary Table S1 for details about chemical features) is distributed to all the hospital buildings after additional filtration and in continuous disinfection with in situ generated chlorine dioxide (Grundfos). Chlorine dioxide concentration in cold water is kept at a residual level of 0.1–0.2 mg/L. 

Water piping is mainly made of crimped AISI 316L stainless steel for risers and return loop and PP-r (random polypropylene) plastic for the end distribution from the risers to the outlets of each room.

The Plate drinking water distribution system is made of risers that bring water to each floor and of horizontal loops that distribute water to all the outlets. The Plate is equipped with three hot water production units (named M1, M2, SO). In each unit, hot water is produced on a plate heat exchanger and is accumulated into two in parallel 5000 l storage tanks. A mixing valve assures that hot water is distributed at the right temperature (see below). M1 and M2 work in parallel, and each of them is proportioned to feed all the main building in case of failure of the other one. SO only feeds the staff changing room at level 1 and the morgue. Both M1 and M2 hot water heaters provide water for the main loop at level 1, from which the risers up to level 3 depart. The recirculation loop at level 3 collects all the flows in the riser and directs them toward both the M1 and M2 hot water production units.

Each tower is equipped with an independent domestic hot water production unit (named T1 to T7) that feeds the tower itself and part of the connecting building with the towers that precede and follow. The hot water production unit, placed at level 0, feeds a loop placed at level 1 that brings water to the risers up to level 6. The main recirculation loop collects the water from the risers on level 5. Each hot water loop also supplies half of the connection buildings (5–6 rooms for each floor) between towers at levels 1 and 2. The recirculation loop for these connection buildings is close to level 2 on the main pipe of the recirculation loop that arrives from level 5.

The hot water production stations were originally designed and realized to carry out thermal shock at 75 °C along all the piping system. For this reason, each room (patient rooms, surgery rooms, ICUs, nursing rooms, staff changing rooms, visitor restrooms, etc.) is equipped with a mixing valve to avoid scalding. Within all the ten years of monitoring herein described, water was kept, thanks to an electronic control system, at 50–55 °C in the storage tanks and at 48 °C after the mixing valves.

Hot water is softened to reach 15 °f and then treated with monochloramine produced and dosed by a patented system (SANIKILL, Sanipur SpA, Flero, Italy) specifically developed to treat domestic hot water. The SANIKILL system synthetizes monochloramine directly into the hot water flow, fine tuning the proper N/Cl ratio, and keeping a constant monochloramine concentration thanks to an on-line Oxidation-Reduction Potential (ORP) probe. Dosing systems are settled to keep monochloramine residual concentration at the outlets between 2 and 3 mg/L. 

Monochloramine and ammonia concentrations are monthly monitored at designed sentinel points within the buildings by on-site analysis with a modified indophenol method (Monochlor F, HACH) performed with a DR900 colorimeter (HACH). 

### 2.3. Water Safety Group and Water Safety Plan

As discussed more thoroughly in the Section 3, even before its opening, the water safety of the hospital has been assured by an interdisciplinary Water Safety Group (WSG) whose first purpose was to implement an ad hoc Water Safety Plan (WSP). Several meetings were organized before the commissioning to reach this goal. After the opening at the end of 2012 until now, the WSG has met at least 4 times a year. 

### 2.4. Sampling and Microbiological Analysis

Starting from November 2011, one year before its opening, the hospital has been checked for the presence of *Legionella* in drinking cold water and domestic hot water systems by the local health authority. 

After the commissioning, an average of 250 samples/year has been regularly collected (one sampling round every 30–60 days) and analyzed by the internal Microbiology and Virology Laboratory (M&V) for the presence of *Legionella* spp. According to WSP requirements, samples were collected from representative points of the domestic hot water distribution systems (e.g., storage tanks, hot water main, recirculating loop, outlets). Starting from 2016, water samples were analyzed also to assess the presence of *Pseudomonas aeruginosa*, *Acinetobacter baumannii*, *Stenotrophomonas maltophilia*, *Fusarium* spp., and *Aspergillus* spp. to fulfill the improved requirements defined by the WSG for a stricter monitoring of water quality in high-risk clinical units hosting immunocompromised patients. 

Samples were collected without flushing and without flaming in accordance with the Italian Guidelines for Legionellosis Prevention. Bacteriological sampling was performed with one-liter sterile bottles supplemented with 1 mL of sodium thiosulphate (10 mg/L) for the neutralization of residual disinfectant.

To ensure the reliability and comparability of the results, a standard procedure for sample collection, transport and storage was established and observed. Samples were delivered to the laboratory for bacteriological analysis immediately after collection. 

Samples were processed according to an internal procedure based on ISO 11731-2:2004 (until 2017) and ISO 11731:2017 (after 2017): 500 mL of water were first vigorously shaken and then filtered by pouring the samples into a sterile 0.45 µm nitrocellulose membrane filter with a 50 mm diameter (International PBI, Milano) using a vacuum source in a biological safety cabinet. The membrane filter underwent acid treatment and washing with PAGE solution. It was then aseptically removed from the holder with sterile filter forceps and directly plated onto Buffered Charcoal Yeast Extract (BCYE) Agar (bioMérieux). Plates were incubated at 35 °C + 2 °C in a humidified incubator with an atmosphere of 2.5% CO_2_. Cell plates were then examined after 72 h of incubation: the negative ones were re-incubated until 10 days after inoculation. The same procedure was followed with the remaining 500 mL of water sample for the analysis of other waterborne pathogens: in this case, the filter membrane was plated onto Tryptic Soy Agar (bioMérieux), incubated at 35 °C + 2 °C in air and examined after 24 h of incubation. The negative ones were re-incubated for a total of 4 days after inoculation for bacteria and of 7 days for fungi.

Growing colonies were then firstly identified by stereomicroscopy and Gram staining. When necessary, presumptive colonies were previously sub-cultured to obtain pure cultures. Finally, 1–3 colonies were directly examined by the MALDI-ToF MS technique using a VITEK^®^MS v2.0 (bioMérieux) analytical procedure according to the manufacturer’s instruction. Isolated bacterial colonies were applied to a single well of a disposable, barcode-labeled target slide (VITEK^®^ MS-DS) using a 1.0 µL loop overlaid with 1.0 µL of a saturated solution of alpha-cyano-4-hydroxycinnamic acid matrix in 50% acetonitrile and 2.5% trifluoroacetic acid (VITEK^®^ MSCHCA) and then air dried. For instrument calibration, an *Escherichia coli* reference strain (ATCC 8739) was transferred to designated wells on the target slide using the procedure described above. The VITEK^®^MS v2.0 system includes an OEM (original equipment manufacturer)-labeled Shimadzu AXIMA Assurance mass spectrometer linked to a reference database, which is referred to as the Knowledge Base. During target interrogation, mass spectra within a range of 2000 to 20,000 Da were recorded in linear positive mode at a laser frequency of 50 Hz. For each interrogation, laser shots at different positions within the target well producing up to 100 mass profiles were summed into a single, raw mass spectrum. The processed (binned) data were used to query the Knowledge Base to determine the taxonomic identity. Since January 2017 until August 2018, the library version was V.3, including six *Legionella* species and subspecies (*L. anisa*, *L. bozemanae*, *L. feelei*, *L. longbeachae*, *L pneumophila* with ssp. *fraseri* and *pneumophila*). Then, the software version V.3.2 was adopted: this library version includes 18 *Legionella* species and subspecies (*L. anisa*, *L. birminghanensis*, *L. bozemanae*, *L. cincinnatiensis*, *L. erythra*, *L. feelei*, *L. jamestowniensis*, *L. jordanis*, *L. londiniensis*, *L. longbeachae*, *L pneumophila* with ssp. *fraseri* and *pneumophila*, *L. rubrilucens*, *L. sainthelensi*, *L. taurinensis*). When *Legionella pneumophila* was identified, serogroups were determined with an agglutination test (*Legionella pneumophila* antisera set, Biogenetics Diagnostics, Italia).

Simplified criteria were then adopted to determine *Legionella* (and other waterborne pathogens) concentration in CFU/L for this unofficial, internal water microbial control. Colonies were manually counted, and the number of colonies obtained was multiplied by 2 (given that 500 mL of water is used for the analysis) in order to obtain the microbial concentration expressed as CFU/L. When higher bacterial concentrations were detected and if the colonies were homogenously distributed on the plate, given that each filter is divided into 145 squares, the value of CFU/L was calculated as follows:

*Cs* = (*a* × 145) × 2

where *Cs* is the value of *Legionella* concentration expressed in CFU/L; *a* is the number of colonies in a square of the filter; 145 is the number of squares in each filter; and 2 refers to the half-liter (500 mL) water-filtered volume.

Starting from 2020, due to the COVID-19 pandemic, the monitoring routine changed by focusing the investigation on *Legionella* and by outsourcing the analysis to a third-party laboratory, which applied the standard ISO 11731:2017 method.

## 3. Results

### 3.1. Water Safety Group, Water Safety Plan and Disinfection Strategy

Given the relevance of Legionellosis and other opportunistic pathogen-driven infections in nosocomial environments, since its opening at the end of 2012, after 7 years of construction works, the water safety of the hospital has been assured by an interdisciplinary Water Safety Group (WSG) composed of the Medical Director, Infection Control Team members, Technical Director and technical staff, Hospital Hygiene Unit, M&V Laboratory Director and proven experts in *Legionella* remediation from external firms. The first purpose of the WSG was to perform an accurate risk assessment and consequently implement a Water Safety Plan (WSP) following the indication of the World Health Organization [4] and national guidelines [29,30]. Several meetings were organized before commissioning to reach this goal. Each decision was submitted to the local health authority for comments and approval. After the opening at the end of 2012, until now, the WSG has met at least four times a year to analyze and discuss monitoring results, update the WSP (at least yearly, as well as in case of significant renovation works or of publication of updated national guidelines, as in 2015) and to take any corrective action required to keep water quality at the highest levels.

The WSG performed and periodically revised an in-depth risk assessment, analyzing the characteristics, function and management of each facility and taking into consideration the features of the water distribution systems. Consequently, they defined, and updated when required, a series of control measures to adopt, a set of protocols to follow, a list of parameters to monitor and a microbial sampling program to implement. As an example, starting from 2016, the WSG suggested, in addition to *Legionella* surveillance, the introduction of a stricter microbial water-monitoring routine by requiring the assessment of *Pseudomonas aeruginosa*, *Acinetobacter baumannii*, *Stenotrophomonas maltophilia*, *Fusarium* spp., *Aspergillus* spp. in high-risk clinical units hosting immunocompromised patients.

Hospital staff were properly trained according to WSP principles and recommendations to actively participate, based on their specific functions, in WSP application by taking care of flushing protocols, maintenance procedures and samples collection.

A crucial step in the definition of reliable control measures to ensure an effective *Legionella* risk management was the choice of a proper strategy for water disinfection. In addition to cold water disinfection with in situ generated chlorine dioxide, the original technical project did not envisage any chemical disinfection for domestic hot water: thermal treatment was proposed for *Legionella* control by keeping the hot water temperature at 65–70 °C in the storage tanks. The risk assessment performed by the WSG highlighted that considering the extension of the hot water distribution systems and given the presence of mixing valves, thermal treatment could be inadequate to effectively avoid *Legionella* proliferation and that the residual chlorine dioxide coming from cold water was obviously insufficient to ensure a proper disinfection.

It was then decided to introduce an additional chemical treatment for domestic hot water: the hypothesis to adopt chlorine dioxide was immediately discarded, given its incompatibility with the PP-r made piping present in the water distribution systems and the unsatisfactory and undesirable outcomes observed in the old building previously hosting the hospital. In that year (2012), as an example, despite the in continuous dosage of chlorine dioxide, 18% of the samples collected (13 out of 72) were positive for *Legionella*.

The WSG decided to adopt an innovative strategy based on monochloramine dosage. 

Monochloramine is in situ produced and dosed proportionally to water consumption and residual concentration, keeping 2–3 mg/L at the outlets. Monochloramine concentration is monthly checked at designed sentinel points within the buildings: the data in Table 1 represent for each year the average concentration of the measurements monthly performed at the hot water return loop of each of the ten domestic hot water distribution systems. These data confirmed that the concentration of monochloramine was maintained within the target range (approximately 2.5 mg/L). Concentration values measured at the outlets (were consistent with the data here summarized.

In addition, ammonia concentration was periodically analyzed: as proved by the data reported in Table 1, the disinfection system ensures an adequate minimization of this chemical species. The average ammonia concentration value was always below the limit value of 0.5 mg/L defined by the Italian legislation.

### 3.2. Legionella Control

The microbial pre-commissioning water sampling performed in 2011 by health authorities highlighted a widespread and consistent microbial contamination: 10 of the 20 samples collected were positive for *Legionella* contamination but it was impossible to properly define the concentration because of the presence of abundant interfering microorganisms. For the other six samples, the concentration of microorganisms was so high to actually preclude any further analysis.

At the end of 2012, before the commissioning, remediation protocols based on a hyperdosage of chlorine dioxide (1 mg/L at distal outlets) and monochloramine (5 mg/L at distal outlets) were performed, respectively, on cold and domestic hot water. The shock treatment was effective in restoring safe microbial conditions of the water systems, zeroing the positivity previously identified: after the treatment, *Legionella* was absent in all the samples collected (147 for domestic hot water and 18 for drinking water).

Since then, the hospital has undergone a strict protocol of microbiological self-checking to confirm the efficacy of the measures adopted for legionellosis risk management. This checking protocol has become increasingly accurate and massive in these 10 years of monitoring: in the last years, more than 300 samples were yearly collected from both drinking and domestic hot water, with an average sampling amount referred to the whole monitoring period of more than 250 samples/year. The results of *Legionella* monitoring are summarized in Table 2.

From 2013 to 2015, none of the samples collected was positive for *Legionella*. 

Starting from 2016, the culture method has been complemented with the MALDI-ToF MS analysis, allowing the identification of all *Legionella* at species level.

From 2016 to 2019, the yearly percentage of positive samples was always below 2% and rarely the concentration exceeded the 100 CFU/L limit stated by the national guidelines. Thanks to the analytical approach adopted, *Legionella* species other than *pneumophila* were also identified (*L. feeleii*, *L. bozemanii*, *L. anisa*).

It is relevant to note that most of the positive samples were isolated from outlets fed by a minor domestic hot water loop lacking continuous disinfection. 

Starting from 2020, due to the emergency status caused by the COVID-19 pandemic, the internal Laboratory of Microbiology and Virology was no longer able to take care of such an extended *Legionella* analysis, which was thus entrusted to an external lab, adopting the standard ISO 11731:2017 method. During 2020 and 2021, the absence of *Legionella* (<100 CFU/L, according to the analytical method limit) was confirmed for all the samples collected. In 2022, only three positive samples were found: all of them were isolated from the already mentioned minor domestic hot water loop lacking a continuous disinfection system.

### 3.3. Additional Microbiological Monitoring

The combined culturing/MALDI-ToF MS analytic approach allowed an improvement of microbiological water monitoring by identifying other waterborne pathogens relevant for nosocomial infections. From 2016 to 2019, the periodical monitoring reports have been enriched for all the samples analyzed by data relative to the detection of the Gram-negative bacteria *Pseudomonas aeruginosa*, *Acinetobacter baumannii* and *Stenotrophomonas maltophilia* and fungi belonging to *Fusarium* and *Aspergillus* genera. Unfortunately, this additional analysis was significantly reduced during 2020, 2021 and 2022 for the same reasons mentioned above.

Table 3 summarizes the outcome of this monitoring: for each of the pathogens considered, the number of positive samples was negligible with respect to the total number of samples analyzed, and no relevant or alarming variations in these microbial populations were generally observed within the seven years of monitoring. Only a slight increase in the number of *P. aeruginosa* positive samples was recorded in domestic hot water, in particular during 2019 (when also for cold water, the number of positive samples was slightly higher) and 2020, even if the percentage of positivity always remained below 10%. The data collected during the last two years, however, suggest a trend reversal, with a reduction in the number of positive samples.

## 4. Discussion

As reported by the ECDC, Legionnaires’ disease does represent an important cause of potentially preventable morbidity and mortality in Europe [34]. Its prevention should rely on an accurate risk evaluation and on the subsequent application of appropriate control measures, especially in the case of settings that host population subgroups more susceptible and at higher risk. This is the case of hospitals, nursing homes and other healthcare facilities. Indeed, as previously mentioned, the last ISS report clearly showed the harmfulness of this illness and of its outcomes in healthcare related-structures [17].

The new European Drinking Water Directive further confirmed the relevance of *Legionella* and its impact on water quality and human health by introducing it as a new parameter to be monitored in all water distribution systems, with a particular focus on water distribution systems of priority buildings. It also stressed the importance of managing this issue with a risk-based approach that allows effective preventive strategies.

The present work describes the experience in Legionnaires’ disease prevention of a new 1000-bed hospital and sharing the encouraging results obtained to prove the effectiveness of the multidisciplinary preventive approach adopted even before its commissioning.

It is indeed well known that construction activities, given their nature and the kind of operations that they may entail, can strongly pose a risk of microbiological contamination the water systems of new buildings (as maintenance works can in pre-existing ones), contributing to the growth and spread of waterborne pathogens in the system itself [35,36]. Not surprisingly, cases of nosocomial Legionellosis attributable to construction or maintenance works have been previously reported [23]. 

For this reason, the Water Safety Group that takes care of *Legionella* risk management started to meet before commissioning to draft a comprehensive Water Safety Plan: they performed a deep evaluation of all the critical issues to be considered when taking care of *Legionella* control in such a complex building and they subsequently defined critical control points and a complete repertoire of preventive and corrective actions to avoid the occurrence of legionellosis cases, according to what reported in previous studies [15,37]. The supervising function of this group, collecting different expertise, plays a crucial role to ensure a constant improvement of risk assessment and a consequent suitable update of the WSP. Similarly, the coordinated activities of a properly trained and informed technical staff (both internal and external) turned out to be essential to effectively put in place preventive and corrective actions. By applying WHO guidelines [4], the management and operating routine adopted since 2012 largely anticipated most of the principles of the risk-based approach invoked by Directive (EU) 2020/2184 and required according to the latest Italian legislation concerning drinking water (D. Lgs. 18/2023).

The choice of the suitable disinfection approach was another key point of the strategy adopted for Legionellosis prevention. The thermal treatment originally suggested in the technical project of the hospital was excluded by the WSG because it was considered ineffective: several studies indeed recently confirmed that thermal treatment cannot be considered a reliable approach to prevent *Legionella* proliferation [38], since it frequently results in regrowth phenomena commonly accompanied by the emergence of viable but nonculturable and thermo-tolerant strains [39,40]. 

Based on these considerations, the adoption of a supplemental and continuous disinfection strategy for domestic hot water was preferred. The severe problems faced in the former city hospital in terms of both disinfection inefficacy and corrosion issues led to the exclusion of chlorine dioxide. Given its high reactivity and its gaseous nature, it has a reduced half-life, especially at high temperature, where its concentration suffers a rapid decrease [41]. It is thus challenging to keep a proper residual disinfection, especially in complex and long water distribution systems. Moreover, its strong oxidizing properties makes it extremely corrosive for both metallic and plastic pipes [33,42,43] with dramatic consequences in terms of leakages and failures [44]. It was even shown that corrosion reactions account for most of chlorine dioxide consumption in hot water systems [45]. 

The WSG thus chose to rely on a disinfection strategy based on the dosage of monochloramine: at that time, despite being internationally approved [4,46] and largely applied for over a century by the US and Canada municipalities as secondary disinfectant for drinking water, it was just at the dawn of its use for *Legionella* control. Nevertheless, it was noteworthy and documented that buildings supplied by municipalities with monochloramine-treated drinking water experienced a reduction in *Legionella* contamination and in hospital-acquired Legionnaires’ disease incidence in comparison with buildings fed by chlorinated water [47,48,49]. Moreover, a study reporting the remarkable results obtained with the first experience of monochloramine application for *Legionella* remediation in the domestic hot water distribution system of a hospital was published just the year before, in 2011. [33].

According to its chemical structure, monochloramine is a weak oxidant [50] that is able to specifically react with some amino acids within microbial proteins [51], thus lethally impairing essential metabolic processes and leading to cell death. Its low reactivity and subsequent enhanced stability enable it to persist within water distribution systems, ensuring a suitable residual disinfection at distal outlets, and to penetrate biofilm [52], quantitatively reaching its deep, actively proliferating layers: here, it effectively exploits its biocidal activity against *Legionella* and other waterborne microorganisms. Given its moderate redox potential, it also ensures compatibility with both plastic and metallic pipes. Indeed, in the case here described, neither pipe failures nor corrosion issues have been reported so far despite the ten-year long uninterrupted treatment.

In terms of disinfection efficacy, our results are in line with what was previously reported by several authors in terms of monochloramine efficacy on *Legionella* control [33,37,53,54,55,56,57]. Although this study cannot prove its remediation properties thanks to the initial shock treatment, based on our observations we can state monochloramine effectiveness in ensuring a long-term control of *Legionella* proliferation, as demonstrated for the first time by a decade-long application experience. This was confirmed by the observation that most of the positive samples identified over ten years were collected from outlets fed by a minor domestic hot water loop lacking monochloramine disinfection. According to its specific risk assessment, the introduction of a dedicated disinfection system has been considered as not a priority so far, and the rare positive cases were handled with minor corrective measures. Nevertheless, in accordance with the aim of control improvement of the WSP, the installation of a continuous disinfection system also on this domestic hot water loop is currently under evaluation. 

The additional microbiological data collected also prove monochloramine effectiveness in keeping other waterborne pathogens of nosocomial relevance under control and ensuring a good microbial quality of water, as already demonstrated by other similar experiences [56,57]. In addition to the waterborne pathogens considered in the present studies, increasing concern is emerging about non-tuberculous mycobacteria (NTMs) and the infections that they can cause [58]. The WSG already took these microorganisms into consideration and included them in the analytical repertoire of microbial monitoring some years ago, but the COVID-19 emergency forced them to abandon the analytical approach based on MALDI-ToF MS and to focus mainly on *Legionella* monitoring. The WSG is now planning to restore the previous analytical approach and to include NTMs in the list of microorganisms to be investigated. 

This parameter can be particularly relevant if considering that some evidence highlighted a reduced susceptibility of these microorganisms to monochloramine [59]. The literature on this topic is actually controversial: indeed, other authors [57], while studying the same monochloramine generator system here adopted, reported by contrast a reduction in NTMs after monochloramine treatment. Similarly, other data [60] seem to suggest that by keeping a proper monochloramine concentration (higher than 2 mg/L, as was the case here described), it is possible to avoid any unintended NTMs proliferation and even eradicate previous contamination. 

As for water safety from a chemical point of view, previous studies demonstrated that the choice of monochloramine ensures the minimization of disinfection by-products typically associated with chlorine-based treatment, such as THM [57,61]. Given that monochloramine is produced by the reaction between chlorine and ammonia precursors, attention should be paid to possible nitrogen-derived by-products like ammonia, nitrites and nitrates, as well as to N-nitrosamines: these by-products are mainly formed when the stoichiometry of the chemical reaction is not properly controlled. It was indeed well documented that when adopting a suitable technology for monochloramine in situ production, all these by-products are produced at negligible levels [37,56,57,61]. The ammonia concentration monitoring here performed confirmed that its concentration was always well below the limit of 0.5 mg/L stated for drinking water by the Italian legislation.

In addition to the choice of a proper disinfection strategy, one of the major strengths of the preventive approach here adopted is the extensive microbial monitoring: the routinely collection and analysis of such a great number of samples enables an extensive and accurate control of the microbiological quality of water and improves our intervention capacity. 

The analytical method here adopted also allows an enhanced sensitivity and thus an improvement with respect to the limits of the traditional standard cultural method. In this way, despite the Italian Guidelines for Prevention and Control of Legionellosis defining a threshold value of 100 CFU/L, more stringent control criteria were internally adopted: even a few units of *Legionella* per liter are taken into consideration and reported, allowing proper and prompt corrective actions. Our method for *Legionella* detection and enumeration ensures a theoretical detection limit of 2 CFU/L, enabling us to carry out remediation measures (like enhancing flushing procedure at critical outlets) well before the contamination can reach alert levels that officially require corrective actions according to the national guidelines.

The choice to adopt an analytical strategy that combines a culture method and MALDI-ToF MS characterization contributed to minimizing *Legionella* related risks. In the last two decades, this protein-based recognition technique has been successfully exploited to easily and quickly identify bacteria and other microorganisms [62,63,64]: it represents a valuable alternative to the conventional phenotypic, biochemical and molecular methods, which is also thanks to its reliability and the remarkable cost-effectiveness.

Previous studies already highlighted all the advantages of choosing this kind of analytical approach proving its effectiveness in the identification of *Legionella* at the species level [65,66]: given the possibility of recognizing a specific peak profile for each species, this approach not only represents a valuable alternative to the traditional identification methods but also broadens the analytical performance, pushing it beyond the simple distinction between *Legionella pneumophila* and *Legionella* spp. and ensuring the possibility of discriminating within the different species of this genus. The MALDI-ToF MS analytical potential was also demonstrated by a recent study describing the promising results obtained in the comparison of the MALDI Biotyper system with culture and gene sequencing techniques for its application in clinical and environmental *Legionella* surveillance [67]. It is indeed crucial to consider that *Legionella* species other than *pneumophila* can be responsible for human infections [68,69]; the ability to identify them is consequently a powerful instrument to recognize and control possible clusters otherwise neglected.

The present study represents one of the first examples of the routine use of this technique to monitor *Legionella* contamination in hospital settings [70]: our experience confirmed it as a reliable and cost-effective tool that is faster and easier with respect to others that allow for species identification.

As demonstrated by the data collected, this analytical approach not only enables an improvement of *Legionella* monitoring but also broadens the microbiological surveillance capability by including the other waterborne opportunistic pathogens previously discussed, increasingly taken into consideration for their health-risk potential [71].

## 5. Conclusions

The WHO reported that when considering the health consequences of waterborne pathogens in the European Union, *Legionella* is responsible for the highest health burden. This is the reason why the New Drinking Water Directive, which came into force on January 2021, introduces the monitoring of this microbiological parameter within the assessment of the potential risks stemming from domestic distribution systems. With the general aim of making the water increasingly safer, it reinforces the principles already stated in the specific guidelines for *Legionella* prevention, stressing the importance of a proper management of the corresponding risk especially for those considered “priority premises”, which is a category that obviously includes hospitals and healthcare-related facilities.

The present study, by reporting the long-term effects of *Legionella* prevention measures adopted by a large and complex hospital since its commissioning, proves indeed the importance of relying on an effective and comprehensive Water Safety Plan. It also confirms the adoption of the continuous dosage of monochloramine as an effective strategy to prevent the proliferation of *Legionella* as well as of other waterborne pathogens of nosocomial concern. The choice of an extensive sampling approach that takes advantage of the analytical potential of the MALDI-ToF MS strategy provides for an accurate and timely control of the microbiological status of water, improving the capacity of safeguarding the health of patients and staff and preserving the hygienic conditions of water distribution systems. This method confirms itself as a powerful tool for the routine microbiological monitoring of water that could thus be further extended to comprise the surveillance of other potentially harmful microorganisms, such as mycobacteria.

## Figures and Tables

**Table 1:** Average monochloramine (NH_2_Cl) and ammonia (NH_4_^+^) concentration values (±standard deviation) expressed in mg/L. Data reported are mean values calculated by the measurements monthly performed at the hot water return loop of each of the ten domestic hot water distribution systems.

Year	NH_2_Cl (mg/L)	NH_4_^+^ (mg/L)
2013	2.57 ± 0.35	0.39 ± 0.13
2014	2.50 ± 0.43	0.43 ± 0.08
2015	2.58 ± 0.62	0.34 ± 0.11
2016	2.66 ± 0.21	0.35 ± 0.14
2017	2.56 ± 0.17	0.35 ± 0.11
2018	2.51 ± 0.27	0.37 ± 0.10
2019	2.51 ± 0.25	0.40 ± 0.06
2020	2.53 ± 0.56	0.37 ± 0.06
2021	2.48 ± 0.55	0.33 ± 0.11
2022	2.49 ± 0.46	0.33 ± 0.10

**Table 2:** *Legionella* monitoring data. For each year of the monitoring period, the number of total samples collected is reported and the distribution between domestic hot water (h) and drinking water (c) samples is specified. The number of samples positive for the presence of *Legionella* spp. (i.e., samples with at least 2 CFU/L) are reported (and expressed as percentage with respect to the total of domestic hot water or cold water samples—value within brackets). The number of positive samples showing a concentration value > 100 CFU/L is also specified (and expressed as percentage with respect to the total of domestic hot water or cold water samples—value within brackets).

Year	Total Samples			Positive Samples (%)	Samples > 100 CFU/L (%)
2013	148	h	138	0 (0.0%)	0 (0.0%)
		c	9	0 (0.0%)	0 (0.0%)
2014	183	h	161	0 (0.0%)	0 (0.0%)
		c	21	0 (0.0%)	0 (0.0%)
2015	191	h	171	0 (0.0%)	0 (0.0%)
		c	20	0 (0.0%)	0 (0.0%)
2016	191	h	169	2 (1.2%)	1 (0.5%)
		c	22	0 (0.0%)	0 (0.0%)
2017	259	h	232	4 (1.7%)	1 (0.4%)
		c	27	0 (0.0%)	0 (0.0%)
2018	300	h	264	5 (1.9%)	3 (1.1%) *
		c	36	1 (2.8%)	1 (2.8%)
2019	255	h	210	3 (1.4%) *	0 (0.0%)
		c	45	0 (0.0%)	0 (0.0%)
2020 **	317	h	251	0 (0.0%)	0 (0.0%)
		c	66	0 (0.0%)	0 (0.0%)
2021 **	363	h	296	0 (0.0%)	0 (0.0%)
		c	67	0 (0.0%)	0 (0.0%)
2022 **	344	h	281	3 (1.1%)	3 (1.1%) *
		c	63	0 (0.0%)	0 (0.0%)

(*) Samples collected from outlets fed by a minor domestic hot water loop lacking chemical treatment). (**) Analysis performed by an external laboratory according to standard ISO 11731:2017 method (negative sample: *Legionella* < 100 UFC/L).

**Table 3:** Other waterborne pathogens monitoring. For each year of the monitoring period, the number of total samples collected is reported and the distribution between domestic hot water (h) and drinking water (c) samples is specified (except for 2022 samples). The number of positive samples (i.e., samples in which the presence of at least 2 CFU/L of the pathogen of interest was detected) is expressed as absolute value and as a percentage with respect to the corresponding total of domestic hot water or cold water samples (value within brackets).

Year	Total Samples			* P. aeruginosa *	* A. baumannii *	* S. maltophilia *	*Fusarium* spp.	*Aspergillus* spp.
2016	135	h	124	1 (0.8%)	0 (0.0%)	4 (3.2%)	0 (0.0%)	1 (0.8%)
		c	11	0 (0.0%)	0 (0.0%)	0 (0.0%)	0 (0.0%)	0 (0.0%)
2017	225	h	196	4 (2.0%)	0 (0.0%)	10 (5.1%)	2 (1.0%)	4 (2.0%)
		c	27	3 (11.1%)	0 (0.0%)	2 (7.4%)	0 (0.0%)	0 (0.0%)
2018	268	h	236	5 (2.1%)	1 (0.4%)	1 (0.4%)	2 (0.8%)	3 (1.3%)
		c	32	0 (0.0%)	0 (0.0%)	0 (0.0%)	0 (0.0%)	0 (0.0%)
2019	307	h	261	18 (6.9%)	0 (0.0%)	8 (3.1%)	1 (0.4%)	6 (2.3%)
		c	46	5 (10.8%)	0 (0.0%)	4 (8.7%)	2 (4.3%)	4 (8.7%)
2020	180	h	147	12 (8.2%)	0 (0.0%)	2 (1.4%)	0 (0.0%)	0 (0.0%)
		c	33	1 (0.7%)	0 (0.0%)	0 (0.0%)	0 (0.0%)	0 (0.0%)
2021	14	h	14	0 (0.0%)	0 (0.0%)	0 (0.0%)	0 (0.0%)	0 (0.0%)
		c	0	0 (0.0%)	0 (0.0%)	0 (0.0%)	0 (0.0%)	0 (0.0%)
2022	76	/		3 (3.9%)	0 (0.0%)	4 (5.3%)	0 (0.0%)	0 (0.0%)

## Data Availability

The data are not publicly available due to hospital internal policy.

## Supplementary Materials

The following supporting information can be downloaded at: https://www.mdpi.com/article/10.3390/microorganisms11071794/s1, Table S1: Physical–chemical properties of municipal drinking water supplied to the Hospital; Table S2: Domestic hot water consumption.
