# Supplementary material for: Water Safety Plan, Monochloramine Disinfection and Extensive Environmental Sampling Effectively Control Legionella and Other Waterborne Pathogens in Nosocomial Settings: The Ten-Year Experience of an Italian Hospital
Source: Microorganisms. 2023 Jul 13;11(7):1794. doi: 10.3390/microorganisms11071794 (PMC10384652; doi:10.3390/microorganisms11071794)
Supplement: Supplementary file 1 [file microorganisms-11-01794-s001.zip › microorganisms-2441084-supplementary.pdf]

SUPPLEMENTARY

Table S1: Physical-chemical properties of municipal drinking water supplied to the Hospital (adapted from data published by water supplier).

Parameter	Value	Reference value	Unit
pH	7.9	6.5-9.5	pH unit
Dissolved solids	209	1500	mg/L
Total hardness	18	15-50	°F
Conductivity 20°C	298	2500	µS/cm
Calcium	47.8		mg/L
Magnesio	14.0		mg/L
Ammonia	< 0.1	0.5	mg/L
Chloride	3	250	mg/L
Sulfate	20	250	mg/L
Potassium	< 0.5		mg/L
Sodium	2	200	mg/L
Arsenic	<1	200	mg/L
Bicarbonate	178		mg/L
Free chlorine	< 0.1	0.2	mg/L
Fluoride	<0.05	1.5	mg/L
Nitrate	4	50	mg/L
Nitrite	<0.05	0.5	mg/L
Manganese	<5	50	µg/L

Table S2: Domestic hot water consumption. For each domestic hot water production unit, mean value of the water monthly consumed is reported, together with relative standard deviation.

Domestic hot water production unit	Water consumption/month
T1	134±2 m^3
T2	114±6 m^3
T3	98±14 m^3
T4	128±21 m^3
T5	118±15 m^3
T6	133±31 m^3
T7	99±12 m^3
SO	43±21 m^3
M1	171±62 m^3
M2	189±99 m^3
